# Larvicidal and adulticidal activities of Purpureocillium lilacinum Secondary Metabolites against the Malaria Vector Anopheles gambiae

**DOI:** 10.21203/rs.3.rs-9556515/v1

**Published:** 2026-05-27

**Authors:** Dongxu Chen, Guodong Niu, Liliana Lai, Cesar Vera Zerpa, Jun Li

**Affiliations:** Florida International University; Florida International University; Florida International University; Florida International University; Florida International University

**Keywords:** insecticide, fungal secondary metabolites, mosquito, malaria, leucinostatin

## Abstract

**Background:**

Malaria control is increasingly threatened by widespread resistance of *Anopheles* mosquitoes to conventional chemical insecticides, highlighting the urgent need for novel and environmentally sustainable vector control agents. The entomopathogenic fungus *Purpureocillium lilacinum* (Thom) Luangsa-ard et al. and its secondary metabolites, including leucinostatins and pulixin, are known to inhibit *Plasmodium* parasites. However, their direct effects on mosquito survival have not been investigated.

**Results:**

Fourth-instar larvae were significantly more susceptible to the crude extract than second-instar larvae, with an approximately four-fold lower LC_90_. Extended fermentation increased larvicidal potency, reducing the LC_90_ from 129 μg/mL to 61 μg/mL. Most fractions showed larvicidal activity, with the two most polar fractions exhibiting significantly greater activity than the crude extract. Purified pulixin and leucinostatins displayed potent larvicidal effects. The LC_90_ values of pulixin and leucinostatin A against fourth-instar larvae were 68 μg/mL and 9 μg/mL, respectively. The LD_90_ of leucinostatin A against adult female mosquitoes was approximately 1 μg per mosquito.

**Conclusion:**

Secondary metabolites from *P. lilacinum* represent promising, cost-effective, and environmentally friendly larvicides against malaria vectors. Because leucinostatin A can inhibit parasite development in adult mosquitoes, kill larvae in aquatic habitats, and kill adult mosquitoes by contact, it may provide a dual-action strategy for blocking malaria transmission.

## 1. Introduction

There were an estimated 282 million malaria cases across 80 malaria-endemic countries in 2024 [1]. Malaria parasites rely on mosquitoes of the genus *Anopheles* for transmission to humans [2]. Approximately two to three dozen *Anopheles* species are considered important vectors of human malaria, among which *An. gambiae* Giles is recognized as one of the most efficient and epidemiologically significant vectors, particularly in Africa [3].

Current malaria vector control depends primarily on two World Health Organization (WHO)-recommended insecticide-based interventions: indoor residual spraying and long-lasting insecticide-treated nets. Six major classes of insecticides are currently used for vector control, including pyrethroids, organochlorines, organophosphates, carbamates, pyrroles, and neonicotinoids [4]. However, the widespread emergence of insecticide resistance has become a major obstacle to effective malaria control. To date, *An. gambiae* populations have evolved resistance to several commonly used insecticides, including pyrethroids, organophosphates, and carbamates [5, 6]. In parallel, the discovery and development of new synthetic insecticides have become increasingly difficult due to rising research, regulatory, and registration costs, as well as growing public concern regarding environmental safety and human health. Together, these challenges underscore the urgent need for safer, sustainable, and mechanistically novel mosquito control agents.

Secondary metabolites are low-molecular-weight compounds produced by plants and microorganisms, typically during specific stages of growth or in response to environmental cues. Although not essential for basic growth and reproduction, these molecules often possess important ecological functions and diverse bioactivities, including roles as pigments, hormones, toxins, and antibiotics. Among microbial sources, entomopathogenic fungi (EPF) have attracted considerable interest because they naturally infect and kill arthropods [7]. EPF are particularly attractive as biocontrol agents because they often exhibit selective toxicity, limited environmental persistence, and can infect hosts through external contact rather than ingestion [8]. Numerous EPF species have shown efficacy against a broad range of agricultural pests, including aphids [9] and stored-grain insects [10], as well as medically important mosquitoes. Fungal genera such as *Metarhizium*, *Beauveria*, *Lecanicillium*, *Aspergillus*, *Nattrassia*, and *Isaria* have been reported as microbial control agents against mosquito genera including *Aedes*, *Anopheles*, *Culex*, and *Culiseta* [11]. Beyond the fungal organisms themselves, fungal secondary metabolites have also emerged as valuable sources of lead compounds for pharmaceutical and agricultural applications, a field accelerated by the development of large and chemically diverse fungal extract libraries [12].

*Purpureocillium lilacinum* (Thom) Luangsa-ard et al. is a well-established entomopathogenic fungus with recognized value as a biological control agent, particularly against plant-parasitic nematodes [13]. Its conidia have also demonstrated pathogenicity against several insect pests, including lace bugs [14], leaf-cutting ants [15], green peach aphids, and fall armyworms [16]. In addition, *P. lilacinum* produces a wide range of secondary metabolites with anticancer, antimicrobial, and insecticidal activities [17]. Among these, leucinostatins are peptide metabolites broadly distributed among *P. lilacinum* isolates and display strong biological activity against yeasts, Gram-positive bacteria, and cancer cells [18]. More recently, several leucinostatins (including A, A1, B, and B1) were shown to block malaria transmission by targeting the mitochondria of *Plasmodium* parasites [19]. Notably, mosquitoes exposed to leucinostatins become refractory to *Plasmodium* infection in the midgut. Leucinostatins also possess nematocidal activity [20, 21], although their insecticidal potential against mosquitoes remains largely unexplored. Furthermore, a recently identified *P. lilacinum* metabolite, 3-amino-7,9-dihydroxy-1-methyl-6H-benzo[c]chromen-6-one (named pulixin), inhibits the interaction between FREP1 and *Plasmodium falciparum*-infected cell lysate and suppresses the proliferation of asexual blood-stage parasites [22].

Given the insecticidal potential of *P. lilacinum* and the urgent need for new biochemical scaffolds for malaria vector control, this study investigated the larvicidal efficacy of its secondary metabolites against the major malaria vector *An. gambiae*. Although previous studies have focused on the antiparasitic effects of leucinostatins and pulixin, their direct effects on mosquito survival and development remain poorly understood. Here, we conducted a systematic toxicological evaluation of crude fungal extracts and their chromatographic fractions to identify the most active larvicidal components. We further assessed purified leucinostatins and pulixin to determine their individual contributions to overall toxicity. By linking fungal secondary metabolism with mosquito larval mortality, this work provides a foundation for the development of next-generation, fungus-derived vector control agents that are both environmentally sustainable and effective against malaria transmission.

## 2. Materials and methods

### 2.1 Rearing of *An. gambiae*

As previously described [23], *An. gambiae* mosquitoes were obtained from BEI Resources. Mosquitoes were maintained in an insectary at 27°C and 80% relative humidity under a 12 h light/12 h dark photoperiod. Adult mosquitoes were provided with 10% sucrose solution ad libitum. Larvae were fed ground KOI fish food until use.

### 2.2 Fungal Fermentation

Fungal extracts were prepared as previously described [12]. Briefly, a *P. lilacinum* seed culture was initiated by inoculating a single fungal colony into 3 mL of malt extract medium containing 10 g/L malt extract, 1 g/L yeast extract, and 0.05 g/L chloramphenicol. The culture was incubated in a shaking incubator at 21°C for 4 days. A sugar solution was then prepared by dissolving 3 g sucrose and 50 mg chloramphenicol in 1 L distilled water, followed by autoclaving for 20 min. Autoclavable mushroom bags equipped with 0.22 μm air-exchange filters were filled with 550 g sterilized Cheerios cereal (General Mills, Golden Valley, MN, USA). The sugar solution, cereal substrate, and fungal seed culture were then combined in each mushroom bag.

### 2.3 Crude Extract Preparation

A total of 15 mushroom bags were divided into three independent batches (n = 5 bags per batch). Batches 1, 2, and 3 were fermented at 21°C for 24, 31, and 45 days, respectively. For each batch, 5 L ethyl acetate (EtOAc) was added to the fermented culture and incubated for 24 h at 21°C with occasional stirring. The supernatant was collected and filtered through a Büchner funnel. The residual pellet was re-extracted with an additional 2.5 L EtOAc. Combined EtOAc extracts were concentrated to dryness using a rotary vacuum evaporator at room temperature, and extract yields were recorded. To remove spores and other insoluble materials, dried extracts were resuspended in EtOAc at a 1:1 (w/v) ratio based on extract mass (e.g., 17 mL EtOAc for 17 g extract) and incubated overnight to generate a saturated solution. Following centrifugation (10,000 × g for 5 min), the supernatant was collected for further separation. The pellet was re-extracted with the same volume of EtOAc to maximize recovery.

### 2.4 Separation and Identification of Active Fractions

The saturated *P. lilacinum* extract was mixed with an equal volume of silica gel (230–400 mesh, 40–63 μm; Sorbent Technologies, Norcross, GA, USA) for dry loading. After drying, the sample–silica mixture was loaded onto a silica gravity column (60 cm × 2.0 cm) packed with 70 g silica gel in hexane. Six fractions (F1–F6) were sequentially eluted, each with four column volumes (approximately 550 mL) of the following solvents: F1, 100% hexane; F2, 100% dichloromethane (DCM); F3, 90:10 DCM:methanol; F4, 80:20 DCM:methanol; F5, 50:50 DCM:methanol; and F6, 100% methanol. Fractions were dried using a rotary vacuum evaporator prior to bioassay.

### 2.5 Bioassay for Larval Stage Sensitivity

Larval mortality bioassays were performed according to World Health Organization (WHO) guidelines with minor modifications [24]. The second batch of crude extract was used to compare susceptibility between larval stages. Ten second-instar or fourth-instar larvae were placed into each well of a six-well plate containing 9.9 mL deionized water. Extracts dissolved in 0.1 mL dimethyl sulfoxide (DMSO) were added to achieve a final DMSO concentration of 1%. Final extract concentrations were 0, 100, 150, 200, and 300 μg/mL for second-instar larvae, and 0, 20, 40, 60, 80, and 100 μg/mL for fourth-instar larvae. Three technical replicates were prepared for each concentration. Plates were maintained in the insectary under standard conditions. Larvae were provided a minimal amount of ground fish food (~ 0.05 mg per larva per day). Mortality was recorded after 24 h. When control mortality was > 0% and < 20%, mortality data were corrected using Abbott’s formula. Bioassays were repeated independently on three separate days.

### 2.6 Bioassays for Fraction Toxicity

Thin-layer chromatography (TLC) was used to evaluate the chemical profiles of crude extracts and fractions F1–F6. Samples were developed in a 90:10 DCM:methanol solvent system and visualized under UV light at 254 nm and 365 nm. For preliminary screening, each fraction was dissolved in DMSO and diluted in deionized water to a final concentration equivalent to the LC50 of its parent crude extract. Three biological replicates were conducted per fraction, with ten fourth-instar larvae per replicate. Mortality was recorded after 24 h. The experiment was independently repeated. To further quantify toxicity, concentration–response assays were subsequently performed. Test concentrations were selected based on the preliminary screening results. Each assay was conducted in triplicate with ten larvae per replicate and repeated on three separate days. Mortality was recorded at 24 h post-exposure.

### 2.7 Larvicidal Activity of Candidate Active Molecules

Several purified compounds present in the more active fractions were selected for evaluation. Pulixin was previously isolated from F3 [22], whereas leucinostatins were isolated from F4 [19]. Because of their relatively high polarity, leucinostatins were also expected to be present in F5 and F6. TLC was therefore performed to confirm their distribution. Pulixin standard was developed alongside F3 using a 90:10 DCM:methanol solvent system. Leucinostatin A was developed alongside F4, F5, and F6 using a 1-butanol:acetic acid:methanol (4:1:1) solvent system. Concentration–response assays were then performed for pulixin, leucinostatin A, leucinostatin B, and a leucinostatin C + D mixture. Compounds were dissolved in DMSO and diluted with water to a final assay volume of 2 mL. Each concentration was tested in triplicate with ten fourth-instar larvae per replicate. Mortality was recorded after 24 h.

### 2.8 Insecticidal activity against adult mosquitoes

The adulticidal activities of fraction F6 and leucinostatin A (LA) were assessed against female *An. gambiae*. Prior to bioassays, mosquitoes were maintained ad libitum on sterile 10% sucrose solution. One-day-old naïve females were cold-anesthetized on ice and separated into groups of 12 individuals per Petri dish. F6 was dissolved in a methanol:acetone (4:6) solvent system at concentrations of 0.5, 1, 2, and 4 μg/μL, whereas LA was prepared at 0.25, 0.5, 1, and 2 μg/μL. A 0.5 μL droplet of each solution was applied to the scutum (notum) of each mosquito, corresponding to final doses of 0.25–2 μg/mosquito for F6 and 0.125–1 μg/mosquito for LA. Control mosquitoes received 0.5 μL of solvent alone. Following treatment, mosquitoes were transferred to 5-oz waxed paper cups (Dart Container, Mason, MI, USA) and provided with 10% sucrose solution. Mortality was recorded 24 h after treatment. All assays were conducted in triplicate and repeated across three independent biological experiments.

### 2.9 Chemical components in F6

The chemical profile of F6 was further characterized using reversed-phase high-performance liquid chromatography (C18-HPLC) on a Shimadzu system (LC-20AD pump, SPD-M20A detector, and FRC-10A fraction collector; Shimadzu, Columbia, MD, USA). Tested compounds (F6, LA + LB, and LC + LD) were dissolved in H_2_O: MeOH 3:7 system at a concentration of 0.5 mg/mL, and approximate 0.5 mL of sample was loaded onto a semi-preparative column (Gemini C18, 250 × 10 mm, 5 μm; Phenomenex, Torrance, CA, USA). Separation was achieved over 30 min at a flow rate of 5 mL/min using a linear gradient of 50% to 60% acetonitrile in water containing 0.1% trifluoroacetic acid.

### 2.10 Statistical Analysis

All statistical analyses and data visualization were performed using GraphPad Prism version 11.0.0 (GraphPad Software). Concentration or dose–response data were analyzed by nonlinear regression to calculate LC_50_ or LD_50_ and LC_90_ or LD_90_ values with 95% confidence intervals. Differences in lethal concentrations or dose among comparable groups were evaluated using the sum-of-squares F-test. One-way analysis of variance (ANOVA) was used to compare larval mortality among crude extracts and derived fractions. When significant differences were detected (p < 0.05), Tukey’s honestly significant difference (HSD) post hoc test was applied for multiple comparisons.

## 3. Results

### 3.1 Yield and Larvicidal Activity of Crude Extracts Produced under Different Fermentation Times

Approximately 2,750 g of Cheerios cereal distributed across five bags per batch was inoculated and incubated at 21°C for 24, 31, or 45 days. The yields of crude extract obtained from the three batches were 17.2, 17.3, and 25.0 g, respectively (Table 1). Despite a 7-day extension in fermentation time, the second batch showed no appreciable increase in yield relative to the first batch (17.3 g vs. 17.2 g). In contrast, extending fermentation to 45 days increased extract yield by 44.5%.

**Figure 1:**
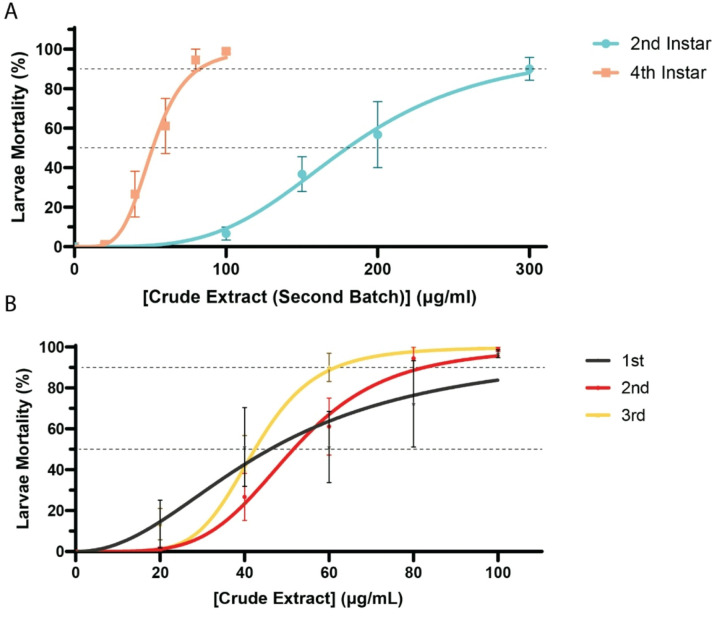
Larvicidal activity of *Purpureocillium lilacinum* crude extracts against second- and fourth-instar *Anopheles gambiae* larvae. **(A)**Concentration–response profiles showing mortality of second- and fourth-instar larvae following exposure to fungal crude extracts. **(B)**Concentration–response curves for three batches of crude extracts tested against fourth-instar larvae. Data are presented as mean ± SEM.

A concentration–response bioassay was performed using the second batch to compare susceptibility between larval stages. Fourth-instar larvae were significantly more sensitive to the crude extract than second-instar larvae (Fig. 1A; Table 2). This difference was evident at both the median lethal concentration (LC_50_: 180.0 μg/mL for second instar vs. 51.56 μg/mL for fourth instar; p < 0.0001) and the higher lethality threshold (LC_90_: 316.6 μg/mL vs. 82.51 μg/mL; p < 0.0001). Based on LC_50_ values, second-instar larvae were approximately 3.9-fold more tolerant than fourth-instar larvae. These findings indicate that larval susceptibility to the fungal extract is strongly influenced by developmental stage.

Concentration–response bioassays were conducted for all three crude extract batches at final concentrations of 0, 20, 40, 60, 80, and 100 μg/mL using fourth-instar *An. gambiae* larvae (Fig. 1B; Table 2). The third preparation exhibited the greatest potency, as indicated by the lowest LC_50_ and LC_90_ values. Differences in LC_50_ values among batches were not statistically significant (p = 0.1601), whereas LC_90_ values differed significantly (p = 0.0264). This pattern suggests that the abundance of major active constituents may be relatively stable across batches, while prolonged fermentation may enhance the accumulation of low abundance but highly potent metabolites that contribute disproportionately to mortality at higher concentrations.

### 3.2 Moderate and Highly Active Insecticidal Fractions

**Figure 2:**
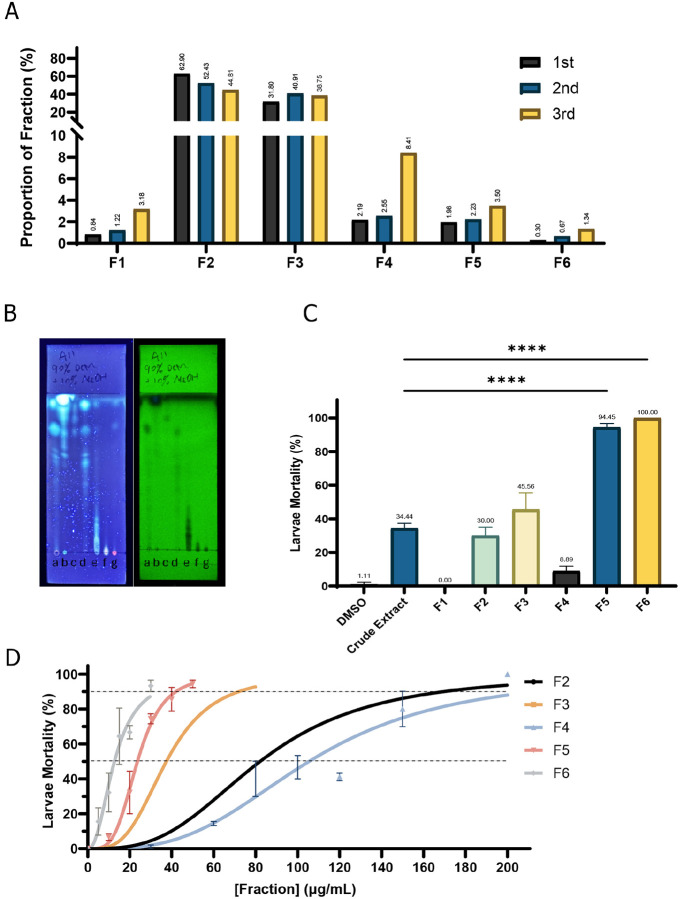
Chemical profiling and comparative larvicidal activity of *Purpureocillium lilacinum* crude extracts and chromatographic fractions. **(A)** Relative mass percentage of each fraction in the crude extracts. **(B)** Thin-layer chromatography (TLC) profiles of crude extract and fractions. Plates were visualized under UV light at 365 nm (left) and 254 nm (right). Lanes a to g represent the crude extract and fractions F1–F6, respectively, all derived from the second batch. Mobile phase: 90:10 dichloromethane (DCM):methanol. **(C)** Larval mortality at 51.6 μg/mL (LC_50_ of the crude extract) for each fraction. ****, p < 0.0001. **(D)** Concentration–response curves of individual fractions. Fractions were derived from the second batch. Each fraction was tested in triplicate with 10 fourth-instar larvae per replicate. Mortality was recorded 24 h after treatment across three independent experiments. Data are presented as mean ± SEM.

Ten milliliters of saturated *P. lilacinum* extract in ethyl acetate was mixed with 10 mL silica gel for dry loading onto a silica gravity column (60 cm × 2.0 cm) packed in hexane. Sequential elution with hexane, DCM, 10% methanol in DCM, 20% methanol in DCM, 50% methanol in DCM, and methanol yielded six fractions (F1–F6). The relative mass contributions of each fraction across the three crude extract preparations were then determined (Fig. 2A).

Fractions F2 and F3 were the dominant components in all preparations, accounting for at least 44.81% and 31.80% of total mass, respectively. Each of the remaining fractions represented less than 8.41% of the extract. With increasing fermentation time, the proportion of F2 declined, whereas the proportions of F1, F4, F5, and F6 increased. Given the reduced LC_90_ values observed in extracts from longer fermentation periods, the enrichment of these fractions may contribute to enhanced larvicidal activity.

Thin-layer chromatography using 10% methanol in DCM revealed progressively shorter migration distances from F1 to F6, consistent with increasing polarity of the constituent compounds (Fig. 2B). To compare biological activity, each fraction was tested at 51.6 μg/mL, corresponding to the LC50 of the second batch crude extract. Mortality was recorded after 24 h of exposure. Fractions F5 and F6 produced significantly higher mortality than the parent crude extract (adjusted p < 0.0001), whereas F2 and F4 showed lower activity and F1 was inactive (Fig. 2C). As a major component of the extract, F3 displayed larvicidal activity comparable to that of the crude extract.

Concentration–response analyses further confirmed these differences (Fig. 2D; Table 3). Fractions F5 and F6 exhibited lower LC_50_ values (~ 23 μg/mL and ~ 13 μg/mL, respectively) than the crude extract (52 μg/mL), and both also showed lower LC_90_ values. Fraction F3 displayed an LC_50_ of approximately 37 μg/mL. In contrast, F2 and F4 showed significantly lower larvicidal activity than the crude extract. Overall, larvicidal potency ranked as follows: F6 > F5 > F3 > crude extract > F2 > F4 > F1. Collectively, these results indicate that the activity of the crude extract reflects both the relative abundance and intrinsic potency of its constituent fractions.

### 3.3 Leucinostatins and Pulixin Exhibit Potent Larvicidal Activity

**Figure 3:**
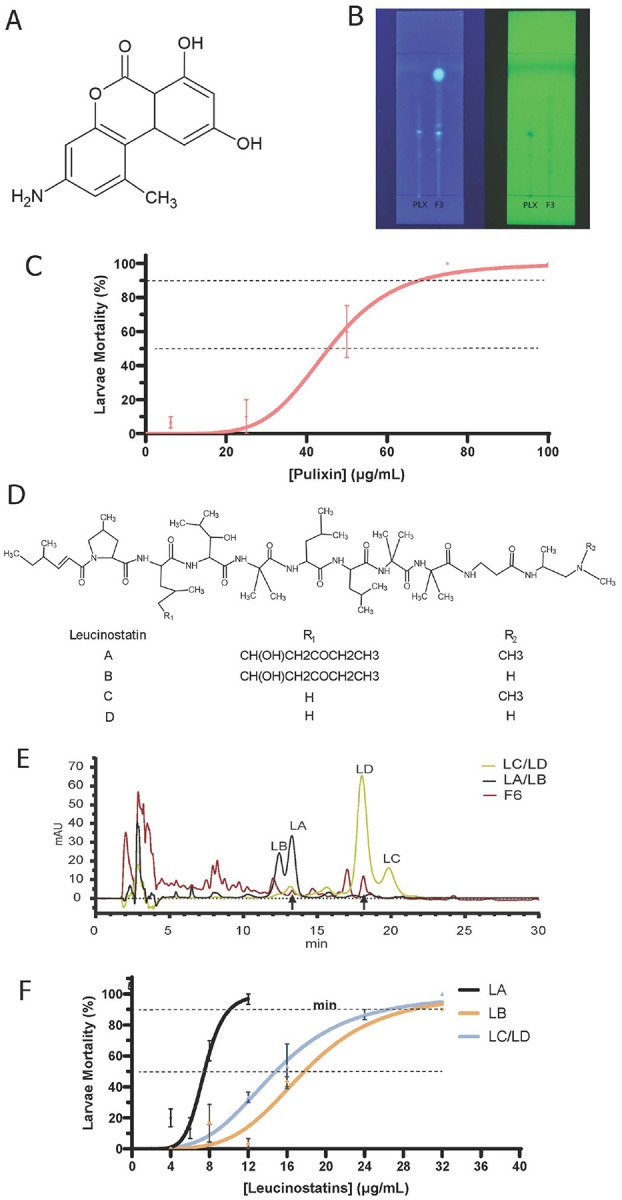
Chemical profiles and concentration–response analyses of pulixin and leucinostatins. **(A)** Structure of pulixin**; (B)** TLC analysis of pulixin (PLX) and fraction F3. Plates were developed in 90:10 DCM:methanol and visualized under UV light at 365 nm (left) and 254 nm (right). **(C)** Concentration–response curves of pulixin against fourth-instar larvae. Assays were conducted in 12-well plates containing 2 mL aqueous solution with 10 larvae per well. Each concentration was tested in triplicate, and mortality was recorded after 24 h. **(D)** Structure of four leucinostatins; **(E)** HPLC profile of leucinostatins and F6. Chromatographic separation was performed using a Gemini C18 column with a linear gradient of 50% to 60% acetonitrile (0.1% TFA) over 30 min at a flow rate of 5 mL/min. **(F)** Concentration–response curves of leucinostatins against fourth-instar larvae.

Several compounds have previously been isolated from *P. lilacinum* [19, 22]. To confirm the presence of pulixin (Fig. 3A) (PLX) and leucinostatins (Fig. 3D) in the active fractions, comparative TLC analyses were performed. Using a 10% methanol in DCM solvent system, purified PLX produced a distinct UV-absorbing spot at 254 nm with corresponding fluorescence at 365 nm and a retention factor (Rf) of approximately 0.5. A matching signal was detected in F3 at the same Rf (Fig. 3B), confirming the presence of PLX. However, F3 also contained additional compounds, including a strongly fluorescent major component with (~ 0.9), indicating that it is chemically complex.

Purified PLX was therefore evaluated independently in larval bioassays. PLX displayed clear larvicidal activity, with LC_50_ and LC_90_ values of 45.53 μg/mL and 68.03 μg/mL, respectively (Fig. 3C; Table 4). Its potency was not significantly different from that of fraction F3 (p > 0.1), suggesting that PLX is a major contributor to the activity of this fraction. HPLC was used to analyze leucinostatin A (LA) and fractions F6 (Fig. 3E). The F6 contained the peaks of LA and LC, consistent with our previous study [19]. Additional UV-absorbing bands indicated multiple constituents.

**(A)** Structure of pulixin; **(B)** TLC analysis of pulixin (PLX) and fraction F3. Plates were developed in 90:10 DCM:methanol and visualized under UV light at 365 nm (left) and 254 nm (right). **(C)** Concentration–response curves of pulixin against fourth-instar larvae. Assays were conducted in 12-well plates containing 2 mL aqueous solution with 10 larvae per well. Each concentration was tested in triplicate, and mortality was recorded after 24 h. **(D)** Structure of four leucinostatins; **(E)** HPLC profile of leucinostatins and F6. Chromatographic separation was performed using a Gemini C18 column with a linear gradient of 50% to 60% acetonitrile (0.1% TFA) over 30 min at a flow rate of 5 mL/min. **(F)** Concentration–response curves of leucinostatins against fourth-instar larvae.

Larvicidal activity assays demonstrated that leucinostatins were highly active against *An. gambiae* larvae (Fig. 3F; Table 4). LA was the most potent compound tested, with an LC_50_ of 7.476 μg/mL, significantly lower than that of leucinostatin B (LB; 17.81 μg/mL) and the leucinostatin C + D mixture (14.83 μg/mL) (p < 0.0001). Similarly, LC_90_ values for LA, LB, and the C + D mixture were 9.278, 19.90, and 22.63 μg/mL, respectively, and differed significantly among treatments (p < 0.0001). These findings identify leucinostatins, particularly leucinostatin A, as the most potent larvicidal metabolites characterized in this study.

### 3.4 Adulticidal Activity of leucinostatin A and F6

**Figure 4:**
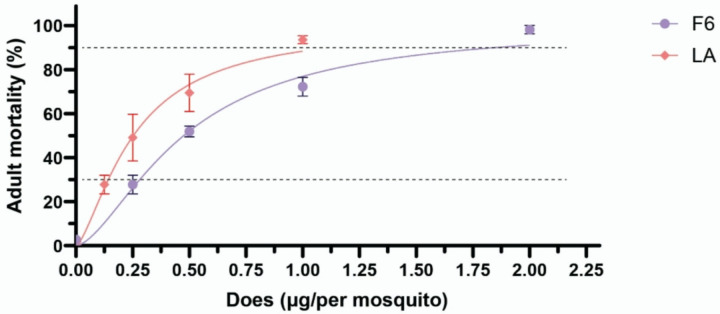
Dose-response curve of F6 and leucinostatin A against adult female mosquitoes. Each dose was tested in triplicate with 12 one-day-old naïve female adult per replicate. Mortality was recorded 24 h after treatment across three independent experiments. Data are presented as mean ± SEM.

Dose-response relationships were determined for LA and F6 against female adult mosquitoes (Fig. 4; Table 5). The LD_50_ values for LA and F6 were about 0.25 μg and 0.48 μg per mosquito, respectively, representing a significant difference (p < 0.0001). While, LD_90_ of LA and F6, 1.122 μg and 1.849 μg respectively were not significantly different (p > 0.05).

## 4. Discussion

Control of agricultural and public health pests has long relied on synthetic insecticides. However, the widespread emergence of insecticide resistance has reduced the durability of many conventional mosquito control tools [25, 26]. This has created an urgent need for new bioactive agents with distinct modes of action, favorable environmental profiles, and compatibility with integrated vector management. In this context, our findings identify *P. lilacinum* as a promising source of insecticidal secondary metabolites active against *An. gambiae*.

Previous work on *P. lilacinum* metabolites focused primarily on their activity against *Plasmodium* [19, 22, 27]. The present study expands their biological relevance by demonstrating direct toxicity to the mosquito vector across multiple life stages. The consistent activity observed in crude extracts, enriched fractions, and purified compounds indicates that this fungus produces several chemically distinct metabolites with mosquitocidal potential.

A notable finding was the greater susceptibility of fourth-instar larvae relative to second-instar larvae. Although early instars are often assumed to be more vulnerable, similar stage-dependent responses have been reported for other fungal metabolites [28]. One explanation is that the active compounds disrupt physiological processes that become especially important during late larval development, such as molting regulation, cuticle restructuring, or preparation for metamorphosis. From an applied standpoint, activity against later instars is advantageous because older larvae are often more difficult to eliminate in operational settings [29, 30].

The crude ethyl acetate extracts of *P. lilacinum* exhibited stronger larvicidal potency against *An. gambiae* than several previously reported fungal extracts [31–33]. These comparisons suggest that *P. lilacinum* contains unusually potent larvicidal metabolites and represents a promising source of new mosquitocidal chemotypes.

Fermentation time also influenced bioactivity. The improved performance of extracts from longer cultures suggests that culture duration alters secondary metabolite composition rather than simply increasing biomass. The enrichment of more active polar fractions after prolonged fermentation is consistent with this interpretation and indicates that fermentation conditions could be optimized to enhance yield of desirable compounds.

Bioassay-guided fractionation further clarified the chemical basis of activity. Pulixin accounted for much of the activity associated with fraction. The strongest overall activity was linked to fraction F6 and the leucinostatin family. However, potency of fractions could not be explained solely by the abundance of identified compounds, suggesting that additional metabolites or synergistic interactions contribute to efficacy.

Among the purified metabolites examined, leucinostatin A emerged as the most potent candidate and retained activity against adult mosquitoes after topical exposure. Information on the arthropod toxicity of leucinostatins remains limited. Previous studies reported that leucinostatin U, Q, and P exhibited moderate nematicidal activity against *Caenorhabditis elegans* but were inactive against *Pratylenchus penetrans* (LC_90_ > 100 μg/mL) [21]. The differences observed among closely related leucinostatin analogs [18, 34] indicate a meaningful structure–activity relationship in which relatively small chemical modifications substantially alter insecticidal potency. This makes the leucinostatin scaffold an attractive starting point for optimization through medicinal chemistry or semisynthetic approaches. Indeed, modification of leucinostatin A changed its activity against parasites [27].

Despite the promising laboratory results, further development is required before practical deployment. Future studies should assess formulation stability, environmental persistence, safety toward non-target organisms, mammalian toxicology, and efficacy under semi-field and field conditions. Identification of molecular targets will also be important for understanding mode of action and predicting potential cross-resistance with existing insecticides.

Malaria control is increasingly challenged by both drug-resistant parasites and insecticide-resistant mosquito populations. Because *P. lilacinum* extracts and the identified leucinostatins can inhibit *Plasmodium* transmission to mosquitoes through external contact while also killing mosquito larvae and adults, they represent a promising dual-action platform for next-generation malaria control strategies.

## 5. Conclusion

This study evaluated the larvicidal activity of *P. lilacinum* crude extracts, derived fractions, and selected bioactive metabolites against the primary malaria vector, *An. gambiae*. Both pulixin and leucinostatins exhibited significant larvicidal effects, with leucinostatin A identified as the most potent compound, showing an LC_50_ of 7.476 μg/mL. LA also had insecticidal activity against adult female mosquitoes, with LD_50_ of 0.2531μg per mosquito. These findings highlight the promise of *P. lilacinum* secondary metabolites as effective, fungus-derived alternatives to conventional insecticides. Because *P. lilacinum* extracts and leucinostatins can both inhibit parasite transmission in mosquitoes and kill mosquito larvae upon exposure, this work provides a strong foundation for the development of next-generation strategies to combat malaria.

## Figures and Tables

**Table 1:** Yield of fungal crude extract at different fermentation times

Preparation	Fermentation Time (Days)	Yield (g)
1st	24	17.2
2nd	31	17.3
3rd	45	25.0

**Table 2:** Larvicidal activity of crude extracts against second- and fourth-instar larvae

Larval Stage	Preparation	LC_50_ (95% CI) (μg/mL)	LC_90_ (95% CI) (μg/mL)	R^2^
4th Instar	1st	46.02 (30.23 to 62.74)	129.6 (75.74 to 431.9)	0.6735
	2nd	51.56 (45.81 to 57.40)	82.51 (69.43 to 102.5)	0.9234
	3rd	42.42 (36.15 to 47.08)	61.51 (45.40 to 77.61)	0.9156
2nd Instar	2nd	180.0 (158.3 to 205.3)	316.6 (244.5 to 465.5)	0.8668

**Table 3:** Larvicidal activity of silica gel column fractions against fourth-instar larvae

Fraction	LC_50_ (95% CI) (μg/mL)	LC_90_ (95% CI) (μg/mL)	R^2^
1	> 200		
2	82.14 (69.85 to 96.92)	169.8 (122.2 to 282.4)	0.8516
3	37.47 (32.18 to 43.51)	72.06 (50.88 to 111.2)	0.8502
4	97.3 (89.08 to 106.4)	213.7 (174.5 to 283.2)	0.9328
5	23.68 (21.41 to 25.89)	42.04 (35.02 to 51.96)	0.949
6	12.72 (10.24 to 15.30)	33.71 (23.08 to 60.43)	0.8477

**Table 4:** Larvicidal activity of pulixin and leucinostatins against fourth-instar Anopheles gambiae larvae

Molecule	LC_50_ (95% CI) (μg/mL)	LC_90_ (95% CI) (μg/mL)	R^2^
Pulixin	45.53 (39.13 to 51.94)	68.03 (50.10 to 85.96)	0.9298
Leucinostatin A	7.476 (6.904 to 8.098)	9.278 (8.122 to 11.90)	0.9049
Leucinostatin B	17.81 (15.46 to 21.27)	19.90 (17.49 to 40.46)	0.8828
Leucinostatin C + D	14.83 (13.47 to 16.31)	22.63 (18.65 to 28.72)	0.9332

**Table 5:** Adulticidal activity of fraction 6 and leucinostatin A against one-day-old female Anopheles gambiae

Compounds	LD_50_ (95% CI) (μg/mosquito)	LD_90_ (95% CI) (μg/mosquito)	R^2^
Fraction 6	0.4750 (0.4119 to 0.5382)	1.849 (1.260 to 2.438)	0.9692
Leucinostatin A	0.2531 (0.1952 to 0.3110)	1.122 (0.4866 to 1.757)	0.9173

## Data Availability

All data generated or analyzed during this study are in this published article.
